# Prevention or no prevention; this is not the question anymore!

**DOI:** 10.1590/2175-8239-JBN-2021-E005

**Published:** 2021-06-04

**Authors:** José Carolino Divino-Filho

**Affiliations:** 1 Karolinska Institutet, CLINTEC, Division of Renal Medicine, Stockholm, Sweden.

There is an old saying that goes: "Prevention is better than remedying". When I arrived at the Lund University Hospital, Sweden, in 1979, I was amazed by the Department of Nephrology's setup, University Hospital and the Swedish Health System. Some people were referred by district doctors to the nephrology outpatient clinic because proteinuria or microscopic hematuria had been detected in urinalyses - one of the medical examination procedures they were subjected to in order to obtain (the youngsters) or renew (the elderly) their driver's license. In Sweden there was already a way to prevent kidney disease through the driver's license!

Nine years later, in a prospective, randomized study by Gutierrez et al. in Stockholm^1^, they showed that more regular outpatient control, without altering protein intake, can delay CKD progression, and the degree of delay is correlated with improved blood pressure control.

Forty-two years have passed since then, and the prevalence of chronic kidney disease (CKD) has reached epidemic levels, today affecting 9.1% of the world population^2^^,^ 3. Hypertension, obesity, diabetes and increased life expectancy are the main reasons why CKD has become a worldwide public health problem.

There are global differences between countries in the incidence and prevalence of CKD, as well as in renal replacement therapies (RRT). These differences are usually associated with several economic aspects, healthcare systems, and the existence of kidney health programs. A recently released book, assessing 51 countries, *Nephrology Worldwide* 4, provides several concrete examples of the effects of socioeconomic disparities on the epidemiology of kidney diseases. CKD is a disease that is prevalent in the young population on the African continent, while in the developed world it predominates in the elderly population. In addition, in most countries there are a large number of patients with CKD diagnosed in the early stages; more than half of the CKD patients in India see a doctor only after developing end-stage renal failure, often requiring dialysis.

Recently (2019), the United States government launched the first order of kidney health strategy, which aims to reduce the number of Americans with kidney failure to 25% over the next decade^5^. This order requires the primary care system to identify people at risk for kidney disease, help these individuals avoid it, and then send these patients to nephrologists to slow the CKD progression.

The retrospective cohort study by Moraes Junior et al. (2021)^6^ estimates the costs of the Brazilian Public Healthcare System (SUS) during the entire pre-dialysis phase (I-IV) and compares them with the amounts spent by the SUS during the dialysis phase (V). The results demonstrate that a pre-dialysis care program can generate an average reduction of R$ 33.023,12, ± R $ 1.676,80 in the average cost for each year of avoided RRT, which is enough to cover the operation of the program. In addition, the authors also demonstrate that RRT costs are very high when compared to the costs of pre-dialysis treatment, even in more advanced CKD stages.

For us, who monitor patients with CKD, this Healthcare Economics study is of great clinical importance: the sooner in the course of CKD patients are diagnosed and followed up in a pre-dialysis program, the longer they will be able to live without the need for RRT. When there is a need to start RRT, they will already be informed of treatment options and be prepared to choose the one that best suits each individual. A patient being followed up in a multidisciplinary and regular manner should be in a better clinical condition to start RRT, and the transition from stage IV to RRT should occur smoothly (with fewer complications and hospitalizations) and, therefore, with lower costs. In fact, the main limitation of this study was that it did not estimate the costs of complications associated with hospitalizations.

The most important message brought by the study is directed to public healthcare managers in Brazil, so that they consider pre-dialysis care as a very important economic option for public healthcare actions and services in the struggle against CKD.

The old adage "Prevention is better than remedying" is wise, while the study by Moraes Junior et al. (2021)^6^ is robust. Therefore, uniting wisdom with science (medicine and economics), in the medium and long run, we should obtain important clinical and socioeconomic results for all those involved in the prevention and treatment of CKD; and even the costs of RRT can be significantly alleviated. Cost studies like this are fundamental and corroborate that prevention is no longer an issue in CKD; now an ACTION is needed from the authorities responsible for Public Healthcare in Brazil and everyone else involved in the treatment of CKD.

## References

[1] Bergström J, Alvestrand A, Bucht H, Gutierrez A (1988). What is the role of controls in an outpatient department on
progression of renal disease ?. Blood Purif.

[2] GBD Chronic Kidney Disease Collaboration (2020). Global, regional, and national burden of chronic kidney disease,
1990-2017: a systematic analysis for the Global Burden of Disease Study
2017. Lancet.

[3] Jager KJ, Kovesdy C, Langham R, Rosenberg M, Jha V, Zoccali C (2019). A single number for advocacy and communication-worldwide more
than 850 million individuals have kidney diseases. Nephrol Dial Transplant.

[4] Moura-Neto JA, Divino-Filho JC, Ronco C (2021). Nephrology Worldwide.

[5] Trump DJ, Peters G, Woolley JT (2019). The American Presidency Project.

[6] Moraes CS, Fernandes NMS, Colugnati FAB (2021). Multidisciplinary treatment for patients with chronic kidney
diseasein pre-dialysis minimizes costs: a four-year retrospective cohort
analysis. Braz J Nephrol.

